# The *E. coli* Global Regulator DksA Reduces Transcription during T4 Infection

**DOI:** 10.3390/v10060308

**Published:** 2018-06-06

**Authors:** Jennifer Patterson-West, Tamara D. James, Llorenç Fernández-Coll, James R. Iben, Kyung Moon, Leslie Knipling, Michael Cashel, Deborah M. Hinton

**Affiliations:** 1 Gene Expression and Regulation Section, Laboratory of Cell and Molecular Biology, National Institute of Diabetes and Digestive and Kidney Diseases, National Institutes of Health, Bethesda, MD 20814, USA; jennifer.west@nih.gov (J.P.-W.); tamara.james@ihs.gov (T.D.J.); kyung.moon@nih.gov (K.M.); lesliek@mail.nih.gov (L.K.); 2 Section on Microbial Regulation, Eunice Kennedy Shriver National Institute of Child Health and Human Development, National Institutes of Health, Bethesda, MD 20814, USA; llorenc.fernandezcoll@nih.gov (L.F.-C.); cashelm@mail.nih.gov (M.C.); 3 Molecular Genomics Core, Eunice Kennedy Shriver National Institute of Child Health and Human Development, National Institutes of Health, Bethesda, MD 20814, USA; james.iben@nih.gov

**Keywords:** bacteriophage T4, DksA, MotA, RNA-seq, transcriptome analysis

## Abstract

Bacteriophage T4 relies on host RNA polymerase to transcribe three promoter classes: early (Pe, requires no viral factors), middle (Pm, requires early proteins MotA and AsiA), and late (Pl, requires middle proteins gp55, gp33, and gp45). Using primer extension, RNA-seq, RT-qPCR, single bursts, and a semi-automated method to document plaque size, we investigated how deletion of DksA or ppGpp, two *E. coli* global transcription regulators, affects T4 infection. Both ppGpp^0^ and Δ*dksA* increase T4 wild type (wt) plaque size. However, ppGpp^0^ does not significantly alter burst size or latent period, and only modestly affects T4 transcript abundance, while Δ*dksA* increases burst size (2-fold) without affecting latent period and increases the levels of several Pe transcripts at 5 min post-infection. In a T4*motA^am^* infection, Δ*dksA* increases plaque size and shortens latent period, and the levels of specific middle RNAs increase due to more transcription from Pe’s that extend into these middle genes. We conclude that DksA lowers T4 early gene expression. Consequently, Δ*dksA* results in a more productive wt infection and ameliorates the poor expression of middle genes in a T4*motA^am^* infection. As DksA does not inhibit Pe transcription in vitro, regulation may be indirect or perhaps requires additional factors.

## 1. Introduction

The integrity and regulation of gene expression is essential for proper cellular function and adaptation. Bacteria have evolved myriad mechanisms to tightly regulate gene expression to ensure that genes are expressed under the correct environmental conditions [1]. Bacteriophages have simultaneously evolved mechanisms for altering gene expression in their host to optimize the expression of genes required for viral proliferation [2,3,4].

Bacterial transcription is performed by an RNA polymerase (RNAP), which consists of five core factors (αI, αII, β, β′, and ω), along with a σ subunit that specifies the transcription start site (reviewed in [5,6]). In *Escherichia coli* (*E. coli*), the primary σ factor, σ^70^, recognizes specific −10 and −35 elements within the promoter sequence and is essential for the transcription of genes needed for exponential growth under nutrient-rich conditions. Efficient transcription requires the interaction of RNAP with promoter DNA to create an “open complex” with a single-stranded region at the transcription start site, initiation of RNA synthesis, promoter clearance, processive transcription elongation, and accurate termination (reviewed in [7]).

*E. coli* promoters use specific activators, repressors, and/or other transcriptional regulators to control initiation, elongation, and termination. While activators and repressors typically interact with specific DNA sequence motifs, DksA is a member of a growing class of global transcription regulators, called secondary (2°) channel proteins, that interact with just RNAP. These proteins, which also include GreA and GreB, all share two superimposable structural domains: a coiled-coil domain that penetrates the 2° channel of RNAP and a globular domain that binds the rim helices of RNAP atop the 2° channel [8]. The acidic residues at the tip of the coiled-coil have protein-specified, DNA context-determined effects on catalysis during transcription initiation as well as elongation and fidelity [9]. These features culminate in activation/inhibition of RNA synthesis (diester formation) as well as diesterase cleavage of backtracked mRNA to rescue transcription arrest [10].

The interaction of DksA with RNAP generates one of two binding sites for the small molecule ppGpp on polymerase [11,12], and in most cases, DksA activity is dependent on ppGpp. While the level of DksA is relatively constant in exponentially growing cells, the concentration of ppGpp increases dramatically under nutrient-poor conditions or other stresses [13,14]. The physiological roles of DksA and ppGpp in *E. coli* gene expression are complicated. DksA/ppGpp inhibition of RNAP initiation from the ribosomal promoter P*rrnB1* is the most widely studied example, where DksA and ppGpp act synergistically to further destabilize already unstable open complexes [12,14,15,16]. In contrast, ppGpp without the help of DksA can inhibit the strong phage lambda pR promoter that forms stable open complexes, not by destabilizing the open complex but rather by inhibiting promoter escape [17]. In other instances, identical, independent, or even opposing regulatory roles have been reported for the two regulators in *E. coli* cells [18]. In addition, ppGpp can bind a variety of proteins with regulatory effects independent of transcription [19]. Furthermore, DksA has a cysteine zinc-finger motif that acts independently of ppGpp to sense oxidative stress [20]. It is noteworthy that GreA and GreB are competitive with DksA in binding to RNAP in order to rescue backtracked, arrested, or paused RNAP, primarily during transcription elongation [21,22].

In a bacteriophage T4 infection, there are three types of T4 promoters (early (Pe), middle (Pm), and late (Pl)) based on the stage of development in which they are active (reviewed in [23]). Pe’s, which contain a strong match for the σ^70^-dependent consensus −10 and −35 elements, do not require any viral factors for activity and are active immediately after infection. Thus, Pe’s successfully compete with host DNA for the same pool of RNAP. Pm’s, which become active ~1 min after infection at 37 °C, require two T4-encoded proteins, MotA and AsiA, to direct RNAP to recognize a specific element, a MotA Box, centered at −30. Importantly, middle genes are also expressed through the extension of early transcription from Pe’s into downstream middle genes. Finally, Pl’s become active ~5–7 min after infection at 37 °C. Pl’s harbor a novel −10 sequence known as the late TATA box and require the T4 proteins gp55 (late sigma factor) and gp33 (co-activator) for activity. In addition, late transcription requires active T4 DNA replication through T4 protein gp45, which is the replication sliding clamp that also serves as an enhancer for Pl’s [24]. As a further complication, phage T4 DNA is modified by glucosylated, hydroxymethyl 5-cytosine, a moiety that can alter RNAP activity as well as the interactions of factors that normally function with unmodified DNA templates.

In this study, we report the first global transcriptome analyses of T4 wild type (wt) and a T4 *motA* knockdown (T4*motA^am^*) infections of a wild-type laboratory strain and a *dksA* mutant host strain, and we investigate the effects of a DksA or ppGpp deletion on T4 growth and transcription. We demonstrate that a deletion of either DksA or ppGpp results in significantly larger plaques for either T4 wt or T4*motA^am^*. Infections in a ppGpp^0^ host have a marginal effect on burst size, latent period, or phage transcript abundance. However, a deletion of DksA results in a 2-fold increase in T4 wt burst size and increased levels of several Pe RNAs. As the T4 activator MotA is required for Pm activation, a T4 *motA* knockdown results in poor phage growth. Our transcriptome analyses reveal that middle genes are affected differentially, ranging from slight to severe inhibition of gene expression. We show that the absence of DksA in the T4 *motA* knockdown background also increases transcription from specific Pe’s. This increase ameliorates the poor growth of T4*motA^am^* by increasing expression of downstream middle genes needed for replication, recombination, and late transcription. As we do not observe an effect of DksA on Pe transcription in a purified in vitro transcription system, we speculate that DksA decreases T4 early transcription through other changes present in the infected Δ*dksA* host or by using additional host/phage factors.

## 2. Materials and Methods

### 2.1. Phage and Strains

T4D^+^ wt and T4*amG1* (T4*motA^am^*) [25] were used for infections. BL21(DE3) (F^–^ *ompT gal dcm lon hsdS_B_*(*r_B_^−^ m_B_^−^*) λ(DE3 (*lacI lacUV5-T7p07* *ind1 sam7 nin5*)) (*malB*^+^)_K-12_(λ^S^)) [26] and B606 (alias REL606), an ancestral B strain of R. Lenski (str^r^, r^–^m^−^, T6^r^, ara^–^) [27] were the parental strains used in this study. Here, strains that bear the functional alleles at the *dksA*, *relA*, and *spoT* loci are referred to as wild type. B606 ∆*dksA::kan* (B606 ∆*dksA*) was constructed by P1 transduction using a K-12 lysate containing the ∆*dksA::kan* insertion [28]. B606 ppGpp^0^, an isogenic mutant strain that is unable to generate ppGpp, was constructed by introducing a ∆*relA::kan* insertion followed by a ∆*spoT::cat* insertion as previously described [29]. Briefly, the d*ksA*, *relA and spoT* mutants were constructed by P1 transduction with *E. coli* K-12 donor strains and the recipient B606 strain. The ∆*dksA::kan* insertion recombinant in B606 grows on LB but not on M9 glucose minimal, like the MG1655 K-12 sister strain. Transduction of the Δ*relA::kan* recombinant allele into B606 was phenotypically verified by loss of resistance to 3-aminotriazole as well as the absence of ppGpp accumulation after adding 1 mg/mL serine hydroxamate. The B606 Δ*relA::kan ΔspoT::cat* double deletion construct was made by introducing the Δ*relA* mutant first, then transduction of the *ΔspoT* mutant since the *ΔspoT relA*^+^ combination is not viable owing to excessive ppGpp levels. Features of the double mutant again mimic those of the K-12 sister strain and are unable to grow on minimal medium without amino acid supplementation.

### 2.2. Quantification of Plaque Size

BL21(DE3), B606, B606 ∆*dksA*, and B606 ppGpp^0^ were freshly streaked out on Lennox L Broth (LB; Quality Biological (Gaithersburg, MD, USA): 5 g/L sodium chloride, 10 g/L tryptone, and 5 g/L yeast extract) plates and grown at 37 °C overnight. For B606 ∆*dksA*, a final concentration of 3 μg/mL adenine was added to plates to improve growth for single colony selection. From a single colony, cultures were grown overnight in LB at 32 °C. For plating, cultures were diluted in fresh LB to an OD_600_ ~ 0.1 and grown to early-log phase (OD_600_ ~ 0.3) at 32 °C. Cells were then transferred to ice until ready to titer. For each titer, ~50 to 100 phages in 200 μL LB were incubated with an equal volume of culture for 5 min, followed by 3 mL of 0.65% (*w*/*v*) Drake Top agar at 48 °C [30], and then plated on 1.5% (*w*/*v*) LB agar. Plates were incubated at 37 °C overnight.

Digital images of each plate were documented using a Protein Simple Molecular Imager Gel Doc (San Jose, CA, USA). TIFF image files were converted to JPEG files with 256 gray levels (0–255) and 650 × 515 pixels resolution in ImageJ [31]. Plaque size was then measured by particle analysis in pixels squared [32]. Individual plaque area measurements were uploaded into R studio [33] and density plots for each replicate were generated using ggplot2 [34]. Density plots provided a visual representation of the distribution of plaque area measurements for each sample.

### 2.3. Burst Size Analysis

Burst size experiments were performed as previously described [35] for single-step growth experiments with the following modifications. B606, B606 ∆*dksA*, and B606 ppGpp^0^ were grown overnight at 32 °C from a single colony, diluted to an OD_600_ ~ 0.1 in LB, and then cultured to mid-log phase (OD_600_ ~ 0.5) at 37 °C. For all conditions, B606 wt was kept on ice and used as the plating strain. For each condition, 900 μL of culture was transferred to a 1.5 mL microcentrifuge tube (pre-warmed to 37 °C) containing either T4 wt or T4*motA^am^* at a multiplicity of infection (MOI) of <0.04. After 10 min, the phage infected sample was diluted 10,000-fold for T4 wt or 1000-fold for T4*motA^am^*. Dilutions were gently vortexed to mix and immediately returned to 37 °C. For pre-burst (≤30 min) and post-burst (35 to 80 min) time points, 100 μL or 10 μL of the diluted phage culture, respectively, was added to 100 μL of B606 wt. This was immediately followed by the addition of 3 mL of 0.65% (*w*/*v*) Drake Top agar (at 50 °C) and plating on 1.5% (*w*/*v*) LB agar. Plates were incubated overnight at 37 °C. Burst size was calculated by dividing the average number of plaques for the post-burst time points (50 to 70 min for T4 wt infection) by the average number of plaques for pre-burst time points (15 to 30 min for T4 wt infections). The latent period is defined as the last time point post-infection for which the relative titer remains less than or equal to 2.

### 2.4. Purification of RNA

B606, B606 ∆*dksA*, and B606 ppGpp^0^ were grown at 37 °C to early/mid log phase (OD600 ~ 0.4), then infected at an MOI of 10 with either T4 wt or T4*motA^am^*. RNA was isolated at 1, 5, or 12 min post-infection using method II [36]. Briefly, T4 infections were rapidly stopped by mixing samples with a frozen solution containing 100 μg/mL chloramphenicol, cells were lysed by treatment with hot phenol, nucleic acid was precipitated with ethanol, the isolated nucleic acid was treated with DNase, and RNA was isolated by phenol extraction followed by ethanol precipitation.

Total RNA used for RNA-seq was assessed on a Bioanalyzer using the Agilent RNA 6000 Nano Kit (Santa Clara, CA, USA) to evaluate the quality of samples; all samples used for library preparation had an RNA integrity number (RIN) of 9.3 or better [37]. The quality of total RNA used for real-time quantitative polymerase chain reaction (RT-qPCR) and primer extensions was evaluated using a 0.8% (*w*/*v*) agarose gel stained with ethidium bromide.

### 2.5. RNA-seq

Total RNA (2.5 µg) from each sample was treated with a Ribo-Zero rRNA Removal Kit (Gram-Negative Bacteria; Illumina, San Diego, CA, USA) to deplete ribosomal RNAs. A TruSeq Stranded mRNA Library Prep Kit (Illumina, San Diego, CA, USA) was then used for the cDNA library preparation, and the library was sequenced using Illumina MiSeq in order to obtain 100 bp paired end reads.

After trimming, FASTQ files were aligned to *E. coli B str. DE3* (NC_012971.2) as a reference genome using STAR v2.5.2 retaining unmapped reads [38]. Unmapped reads, presumably stemming from the T4 phage genome, were then mapped to the T4 reference genome (NC_000866.4). In both cases, default alignment behavior was altered with the following arguments: --outFilterScoreMinOverLread 0 --outFilterMatchNmin 30 --outFilterMatchNminOverLread 0 --clip3pAdapterSeq AGATCGGAAGAGCGTCGTGTA --alignIntronMax 1. RNA gene counts in both reference genomes were then quantified using the same NCBI gene definitions utilized in mapping index construction using the subread featureCounts v1.4.6-p3 package [39]. Differential expression between samples and control was represented as a fold change, and genes with both a fold change ≥2 and *p* value ≤ 0.05 were considered significant. RNA-seq data is available in the National Center for Biotechnology Information (NCBI) database (GEO number GSE111808) and in Table S1.

### 2.6. Primer Extension Analyses

Primer extension was performed using Avian Myeoloblastosis Virus (AMV) reverse transcriptase (Life Sciences, Inc., St. Petersburg, FL, USA) as previously described [40,41,42], using the RNA sample (2 μg for the 1 min time points, 4 μg for other time points) and 1 pmol of 5′-^32^P-labeled primer. Unless otherwise indicated, oligonucleotides annealed ~100 nucleotides (nt) downstream of the predicted start site of the promoter. Primer sequences are available upon request. Primer extension products were separated on 5% (*w*/*v*) denaturing polyacrylamide (19:1, acrylamide:bis) gels and visualized by autoradiography. Product levels were quantified using a GS-800 calibrated densitometer (Bio-Rad, Inc., Hercules, CA, USA) and Quantity One Software (Bio-Rad, Inc.)

### 2.7. RT-qPCR

RT-qPCR was used to validate RNA-seq data. Briefly, RNA samples were collected under the same conditions used for RNA-seq, and complementary DNA (cDNA) was generated using a High Capacity cDNA Reverse Transcription Kit (Applied Biosystems, Foster City, CA, USA). Target genes were amplified with SYBR Green PCR Master Mix (Applied Biosystems) in a LightCycler 480 instrument (Roche Molecular Diagnostics, Pleasanton, CA, USA). Relative levels of gene expression were determined with the comparative C_T_ method, also known as 2^-ΔΔ*C*t^ [43]. Genes *nrdA* (B606 ∆*dksA* vs. B606 wt) and *61.4* (T4*motA^am^* vs. T4 wt) that appeared unaffected in the RNA-seq data were used as internal controls. Primers used in this study were designed using Primer3web [44,45]; primer sequences are available upon request.

### 2.8. Proteins

Core RNAP was purchased from New England Biolabs, and σ^70^ [46], MotA [47], and AsiA [48] were purified as previously described. DksA containing a C-terminal His_6_ tag was isolated and purified from CF9413:MG1655 (λDE3)/pHM1501 [49] grown in 500 mL of LB (Quality Biological) containing 50 µg/mL carbenicillin at 37 °C with shaking until mid-log phase. At an OD_600_ of ~0.35, protein synthesis was induced by the addition of 0.2 mM IPTG (final concentration) for 2 h with shaking. Cells were harvested by centrifugation at 13,000× *g* for 10 min. The following procedures were performed on ice or at 4 °C. Pellets were resuspended in 12 mL of lysis buffer (40 mM Tris-HCl (pH 8.0), 300 mM NaCl, 5 mM imidazole, 2 mM 2-mercaptoethanol, 0.1 mM benzamidine; 2–5 mL per gram wet weight) and lysed by sonication. Clarified supernatant was obtained by centrifugation at 15,000× *g* for 30 min and added to 2 mL of a 50% slurry of equilibrated Ni-NTA agarose (QIAGEN, Hilden, Germany) in lysis buffer and rocked gently overnight. The lysate/Ni-NTA mixture was loaded onto a Bio-Rad 10 mL disposable column, washed twice with five bed volumes of wash buffer (40 mM Tris-HCl (pH 8.0), 300 mM NaCl, 10 mM imidazole, 2 mM 2-mercaptoethanol), and then with 4 mL each of wash buffer containing increasing imidazole concentrations: 60 mM, 150 mM, and 250 mM. Although all imidazole fractions contained DksA, the 250 mM fraction was highly purified (Figure S1). This fraction was pooled and dialyzed in DksA Storage Buffer (25 mM Tris-HCl (pH 8.0), 100 mM NaCl, 0.5 mM ethylenediaminetetraacetic acid (EDTA), 50% glycerol, 2 mM 2-mercaptoethanol) and stored at −20 °C.

### 2.9. In Vitro Transcription

In order to check the activity of purified DksA, in vitro transcription assays were performed using a supercoiled template DNA that contains the ribosomal promoter, P*rrnB*P1 (*rrnB* sequence −180 to +109) [49] as a positive control or a DNA template, P*S17* (gift of J. Nguyen) that contains a σ^70^-dependent promoter that is not affected by DksA as a negative control. Transcription reactions (10 µL) were assembled with 5 nM DNA, 30 nM RNAP (final concentration; sigma:core ratio of 2.5:1) that had been reconstituted with or without 250 µM ppGpp (final concentration), the indicated concentration of DksA, and transcription buffer (50 mM Tris-acetate (pH 8.0), 10 mM magnesium acetate, 10 mM 2-mercaptoethanol, 90 mM potassium glutamate, 10 µg/mL bovine serum albumin). Solutions were incubated at 37 °C for 10 min before single round transcription was initiated by the addition of heparin (final concentration of 100 μg/mL) and ribonucleoside triphosphates (rNTPs; final concentration of 200 μM each ATP, GTP, CTP and 20 μM (α-^32^P) UTP at 1.7 × 10^4^ dpm/pmol). After incubation for 10 min at 37 °C, reactions were collected on dry ice. A solution (10 μL) containing 10 mM EDTA (pH 7.0), 0.1% bromophenol blue, and 0.1% xylene cyanol in deionized formamide was added. Samples were then heated for 2 min at 95 °C and 10 μL aliquots were electrophoresed on 4% polyacrylamide, 7 M urea denaturing gels run in 1× TBE (Tris-borate-EDTA). Gels were imaged by autoradiography as described above.

For in vitro transcriptions with T4 DNA, T4 genomic DNA was purified from concentrated T4 wt by extraction with phenol, phenol:chloroform:isoamyl alcohol (25:25:1), and then chloroform:isoamyl alcohol (24:1); each extraction step was repeated twice. The purified DNA was subsequently dialyzed against TE buffer, and then treated with T4 DNA ligase (New England Biolabs, Ipswich, MA, USA) in the presence of ATP to seal any intramolecular nicks. Transcription reactions (100 μL) were assembled with 0.06 pmol of T4 DNA, 7.5 pmol pre-reconstituted RNAP (sigma:core ratio of 2.5:1), transcription buffer, and as indicated, 112.5 pmol AsiA, 1.88 pmol MotA and/or 60 pmol DksA. After incubation at 37 °C for 10 min, a single round of transcription was initiated by adding heparin (final concentration of 100 μg/mL) and rNTPs (final concentration of 200 μM each ATP, GTP, CTP, UTP). Samples were incubated for 10 min at 37 °C before collection on dry ice. An independent in vitro transcription reaction was performed as described [40] using pGEX-5X-3 DNA (0.05 pmol, Pharmacia Biotech, Piscataway, NJ, USA), which contains the P*tac* promoter. Before RNA isolation, a 10 μL aliquot of the P*tac* reaction was added as an internal control into each sample, and then the RNA was extracted and purified by phenol extraction/ethanol precipitation as described above. 5′-ends of the RNA were detected by primer extension analyses as described above.

## 3. Results

### 3.1. T4 Infections in the Absence of ppGpp or DksA Produce Larger Plaques

**Figure 1:**
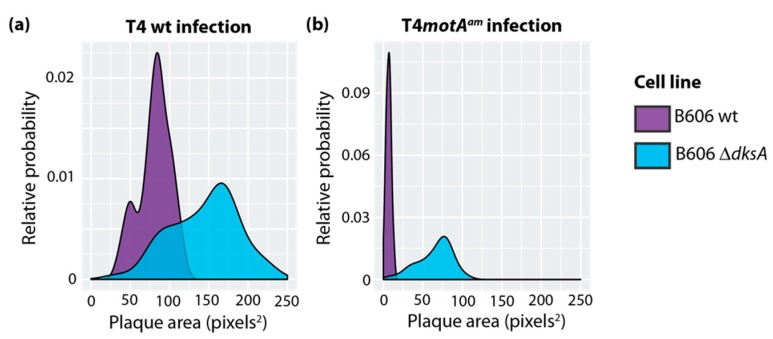
T4 infections in the absence of DksA produce larger plaques. Density plots of plaque size for B606 wild type *(*wt; purple) and B606 ∆*dksA* (blue) infected with either T4 wt (**a**) or T4*motA^am^* (**b**). Plots shown represent one of three biological replicates.

Plaque size can be used as an indirect measure of phage fitness since larger plaques can arise from better phage growth. T4 wt infections of different *E. coli* wt strains typically yield plaques with similar sizes. For example, using two different *E. coli* B strains, B606 wt or BL21(DE3) wt, we observed similar T4 wt plaques with minimal heterogeneity (average size of ~96 pixels^2^ and ~81 pixels^2^, respectively) (Figure S2a). In contrast, T4 wt infections of either B606 ∆*dksA* (Figure 1a) or B606 ppGpp^0^ (Figure S2b) produced broad populations with both larger plaques and increased heterogeneity (average size of ~147 pixels^2^ and ~144 pixels^2^, respectively). An amber mutation in the T4 gene *motA* (T4*motA^am^*) is known to significantly impair T4 infection due to a dramatic decrease in middle gene expression and delayed replication ([25,42]; reviewed in [23]). As expected, a T4*motA^am^* infection produced significantly smaller plaques than a T4 wt infection of the same strain (Figure 1b vs. Figure 1a). Surprisingly, larger T4*motA^am^* plaques were observed with either B606 ∆*dksA* (Figure 1b) or B606 ppGpp^0^ (Figure S2c) (average size of ~65 pixels^2^ and ~39 pixels^2^, respectively) compared to B606 wt (average size of ~7 pixels^2^).

Although B606 ∆*dksA* grows more slowly than wt as cells enter stationary phase, we did not observe a marked difference in growth among wt, B606 ∆*dksA* and B606 ppGpp^0^ during early to mid-log phase, when T4 infects (Figure S3). Consequently, we concluded that the differences in T4 plaque sizes did not arise from significantly different growth rates for the host strains.

### 3.2. Deletion of dksA Results in an Increase in Phage Progeny per Infection

**Figure 2:**
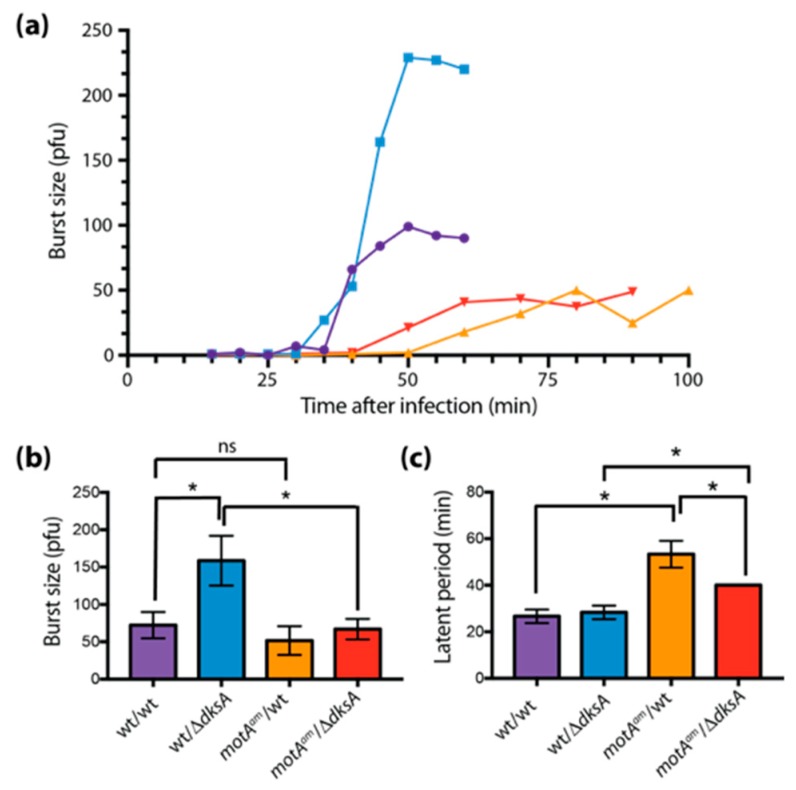
Absence of DksA increases the burst size of a T4 wt infection and decreases the latent period of a T4*motA^am^* infection. (**a**) Representative graph of burst size vs. time after infection; (**b**) burst size, and (**c**) latent period for infections of T4 wt/B606 wt (purple), T4 wt/B606 ∆*dksA* (blue), T4*motA^am^*/B606 wt (orange), and T4*motA^am^*/B606 ∆*dksA* (red). Averages with indicated standard deviations were determined as described in Materials and Methods from three independent replicates. (The error bar on *motA^am^*/∆*dksA* is too small to be visible.) Asterisks (*) indicate significance as determined by an unpaired t test with a *p* value ≤ 0.05. The apparent 5 min difference in latent period of the wt/wt and wt/∆*dksA* infection shown in (**a**) is not statistically significant (see panel (**c**)).

Plaque size is affected by several factors, including the number of phage progeny (i.e., burst size), diffusivity, absorption rate, and length of the latent period [50,51]. To assess whether the larger plaque size observed in the absence of DksA or ppGpp arose from an increase in the number of phage progeny, we performed burst size experiments for T4 wt infections of B606 wt, B606 ∆*dksA*, and B606 ppGpp^0^. We found that the absence of DksA increased the burst size ~2-fold (Figure 2a,b), while the absence of ppGpp had no statistically significant effect (Figure S4). In addition, we observed no drastic change in latent period for any of the infected strains (Figure 2a,c and Figure S4a). We conclude that the increase in plaque size for T4 wt in the absence of DksA can be attributed to an increase in phage progeny per infection. Conversely, the increase in plaque size in the absence of ppGpp is not related to burst size or latent period.

### 3.3. Deletion of dksA Results in a Shorter Latent Period in a T4motA^am^ Infection

Similar burst size experiments were also performed with T4*motA^am^*. In this case, we did not observe a significant difference in burst size for T4*motA^am^* in host wt vs. ∆*dksA* strain (Figure 2a,c). However, in the absence of DksA, there was a 10 min decrease in the latent period, indicating a significantly faster infection (Figure 2a,c).

### 3.4. Global Transcriptome of the T4motA^am^ Infection Reveals that Pm’s Are Differentially Affected

To investigate the effect of DksA on gene expression in a T4 wt or T4*motA^am^* infection, we first collected detailed information on gene expression alterations in a T4*motA^am^* infection of B606 wt. Although extensive work has established that *motA* is required for activation of T4 middle promoters, a global transcriptome analysis of the *motA* regulon has not been reported. It should be noted that while a *motA* deletion is lethal [52], the T4*motA^am^* mutation is not. Nevertheless, there is a dramatic inhibition of transcription from MotA-dependent promoters and a DNA delay phenotype [40,42,52].

We therefore carried out RNA-seq to define the MotA regulon by comparing gene expression in B606 wt cells infected for 5 min at 37 °C with either T4 wt or T4*motA^am^*. At the 5 min infection point, middle genes were highly expressed, early transcription was ending, and late gene expression had initiated (reviewed in [53,54]). Genes, whose expression changed ≥2-fold with a *p* value ≤ 0.05, were considered significant.

**Figure 3:**
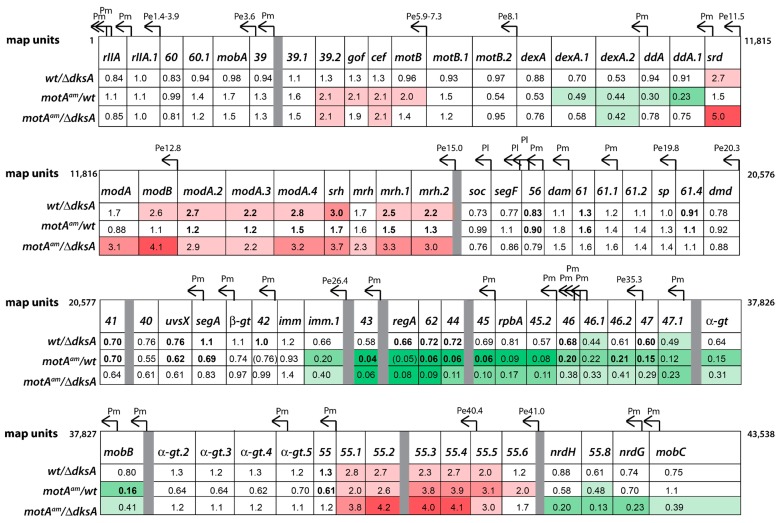

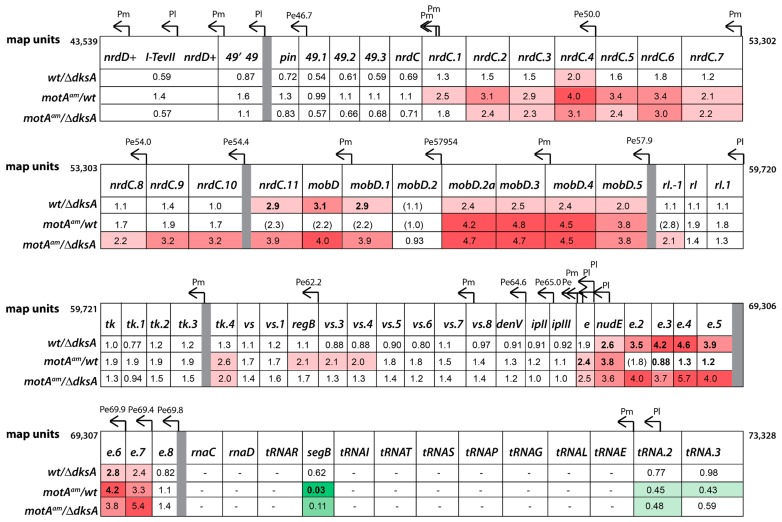

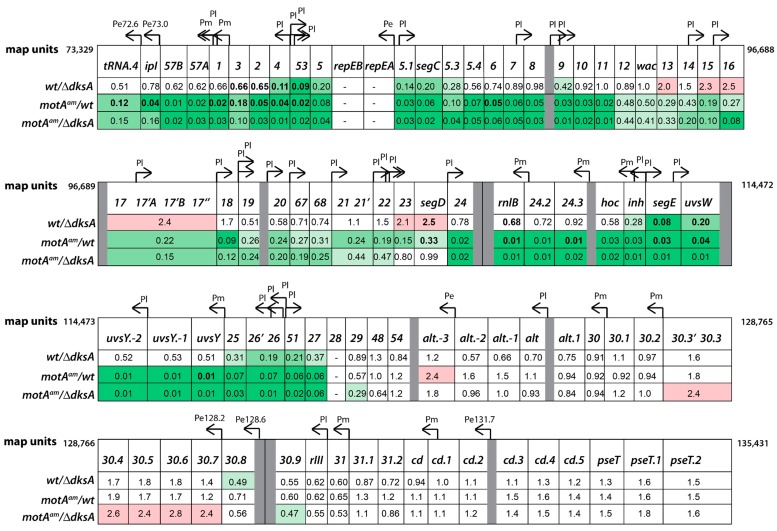

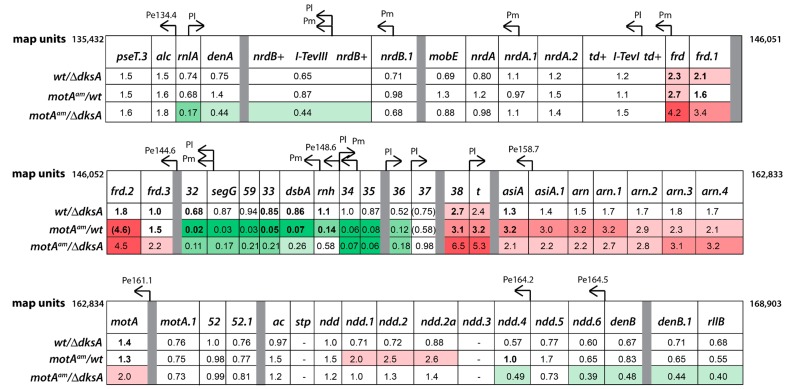
Genomic map of bacteriophage T4 correlated with gene expression data from RNA-seq. Graphical representation of the T4 genome showing the location of annotated genes and promoters as given in [53]. The fold changes from the RNA-seq analyses determined for infections of T4 wt/B606 *dksA* (wt/*dksA*), T4*motA^am^*/B606 wt (*motA^am^/*wt), and T4*motA^am^*/B606 ∆*dksA* (*motA^am^/dksA*) compared to T4 wt/B606 wt are indicated. Gene expression changes are color coded as: increasing expression ≥2.0 with increasing red intensity; no significant change, white; decreasing expression ≤0.5 with increasing green intensity. Values of genes whose expression was checked by real-time quantitative polymerase chain reaction (RT-qPCR) are given in bold; any value that was tested in RT-qPCR analyses but was not consistent is given in parenthesis. Genes without an indicated value had less than 50 mapped reads in all the RNA-seq data samples. The genomic coordinates (map units) for each region are listed at the start and end of that row of genes.

As shown schematically in Figure 3, the T4 genome is generally organized as regions of pre-replicative genes (early and middle) that are transcribed right-to-left and interrupted by regions of late genes that are transcribed left-to-right (reviewed in [53]). In general, early genes encode products needed for middle gene expression and to usurp host processes. However, the functions of many early genes are still unknown. Middle genes encode products needed for the crucial functions of replication, recombination, late gene transcription, and the continuation of host takeover. Late genes mainly encode morphological proteins. Since the majority of the T4 Pe’s, Pm’s, and Pl’s have been identified (reviewed in [23,53]), we generated a detailed diagram of early, middle, and late transcription along the entire T4 genome (Figure 3). Convention within the field has provided designations for Pe’s based on genomic coordinates (e.g., Pe15.0), whereas Pm’s and Pl’s are indicated by the first gene within the resulting transcript (e.g., Pm*dsbA* and Pl*15)*.

Previous global analyses of T4 gene expression using hybridization studies or levels of various phage proteins during a T4*motA^am^* infection have indicated that when MotA-dependent promoters are not activated, expression of some early genes remains high, while late transcription, which is dependent on the middle products gp55, gp33, and gp45, is significantly diminished (reviewed in [54]). However, the expression of middle genes varies. This phenomenon arises in part because the effect of the *motA* knockdown on Pm activation is promoter specific [40,42,55]. In addition, most middle genes are transcribed by both Pm’s and upstream Pe’s (Figure 3). Therefore, the effect of T4*motA^am^* on a particular middle gene also depends on how much transcription from a Pe extends into this gene. While the mechanism for this elongation has not been fully elucidated, it appears to be dependent on processes that stabilize RNA rather than by anti-termination [56,57]. Even though the 3′ ends of many of these transcripts have been identified (indicated as grey bars in Figure 3), these ends are thought to represent the ends of the stable RNA rather than transcription termination sites.

Differential expressions determined by RNA-seq analysis were compared to RT-qPCR assays of 68 genes using RNA isolated 5 min after infection and by primer extension analyses using RNA isolated at 1 and 5 min after infection. One min after infection, early promoters are active, but middle transcription has just initiated [53,54]. A comprehensive summary of our RNA-seq and RT-qPCR analyses is shown in Figure 3. Overall, we observed a strong correlation between the results of the RNA-seq and RT-qPCR analyses (Figure S5a).

Not surprisingly, our RNA-seq/RT-qPCR analyses were consistent with the results from previous studies of T4*motA^am^* infections. Expression of 44% of early genes increased ≥2-fold, while expression of 79% of late genes decreased ≥2-fold (Figure 3). (Genes are classified according to the expression patterns observed in a previous global microarray analysis of a T4 wt infection [58].) Expression of middle genes was mixed, and middle genes that show significant MotA dependence varied in the magnitude of their dependence.

**Figure 4:**
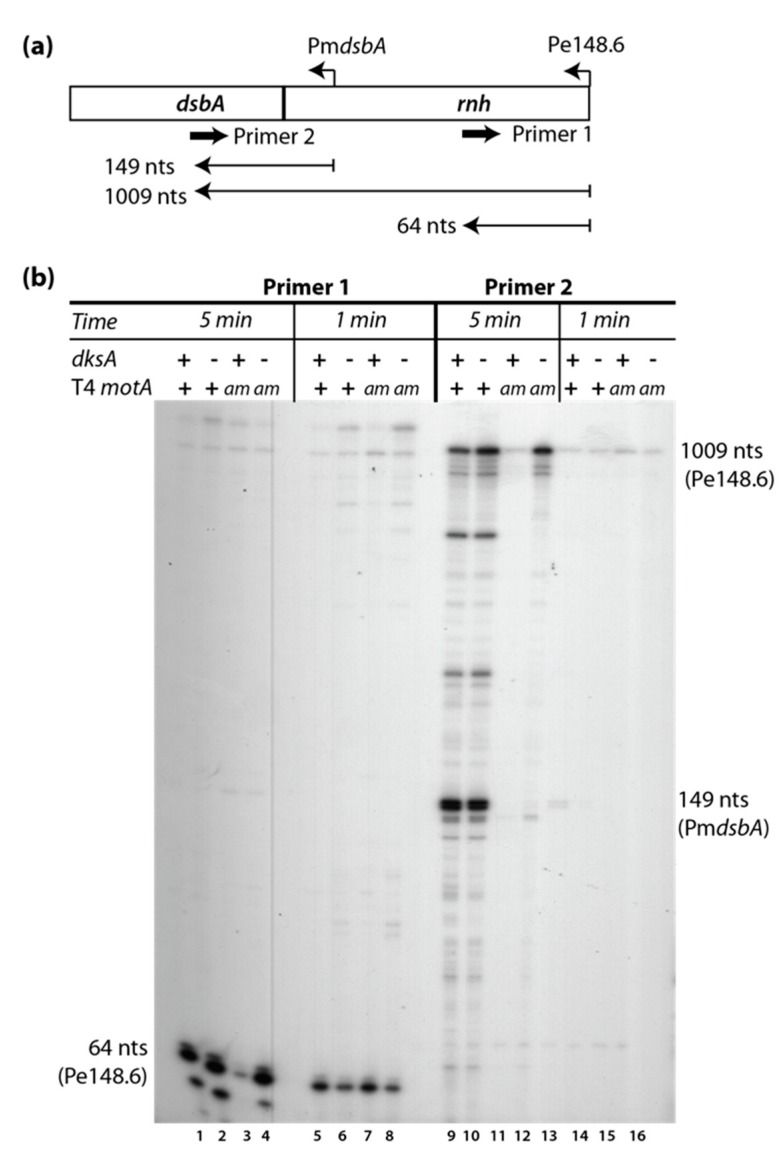
RNA from Pe148.6, which is significantly reduced in a T4*motA^am^* infection of B606 wt at 5 min post-infection, is rescued by a deletion of DksA. (**a**) Schematic showing T4 map units from 150,727 to 149,509 containing genes *rnh* and *dsbA* with the relative positions of the early promoter Pe148.6 and the middle promoter Pm*dsbA* and the primers used for primer extension analyses. Sizes of the expected primer extension products are indicated in nts; (**b**) Representative gel showing the primer extension products from primers indicated in panel (a). RNA was isolated from T4 wt (+) or T4*motA^am^* (*am*) infections of strain B606 wt (+) or B606 Δ*dksA* (−) at 5 or 1 min post-infection.

To confirm that changes in gene expression arose from defects in Pm transcription, primer extensions were performed for several middle genes (Figure S6a). As expected, this analysis confirmed previous results indicating that the *motA^am^* mutation results in a significant decrease in Pm activation. In addition, we also observed lower transcription in the T4*motA^am^* infection that maps to one early promoter, Pe148.6 (Figure S6b). This result is surprising because the classification of this promoter as a Pe is confirmed by its activity 1 min after infection (Figure 4b, lanes 5–8) and because there is no recognizable Pm sequence at this site. However, reduced transcription from Pe148.6 has also been observed in a T4*motA^am^* infection of another cell line [40].

Overall, these analyses provided a “proof of principle” that our RNA-seq analyses correctly reflected the state of T4 transcription 5 min post-infection. However, this study also significantly extended previous work because one can now investigate the effect of the *motA* knockdown on each gene throughout the T4 genome. Interestingly, our results indicate that there did not appear to be any general correlation between the function of a middle gene and its dependence on MotA. As seen in Figure 3, within regions that contain both Pe’s and Pm’s, we observed two sections of T4 genes that are crucial for replication and late transcription and are highly dependent on MotA: (1) the region downstream of Pe35.3 (map units 35,662 to 27,197) that includes gene *43* (DNA polymerase), gene *45* (DNA polymerase clamp and late transcription enhancer), and genes *44*/*62* (clamp loader) and (2) the region downstream of Pe148.6 (map units 150,727 to 146,948) that includes gene *33* (co-activator required for late transcription), *rnh* (RNase H), gene *59* (DNA helicase loader for replication), and gene *32* (single-stranded DNA binding protein). However, other sections with Pe’s and Pm’s, whose genes also encode important replication and late transcription factors, were not significantly MotA-dependent. These included: (1) the region downstream of Pe26.4, Pe20.3, and Pe19.8 (map units 27,044 to 17,935) containing genes *41*/*61* (DNA helicase/primase) and *uvsX* (*recA* analog), and (2) the region downstream of Pe40.4 (map units 41,225 to 39,600) containing gene *55* (sigma factor for late transcription). Because the Pm’s for both these regions (Pm*55* and Pm*uvsX*) are highly dependent on MotA (Figure S6a), we conclude that it is the extension of Pe’s into these regions that compensates for the lack of MotA.

Thus, our analyses indicate that at 5 min post-infection in the *motA* knockdown, the expression of only a subset of middle genes encoding replication/recombination proteins was near wt levels. These include the genes for helicase (gp41), the recombination protein UvsX, EndoVII (gp49) that resolves recombination structures, nucleases gp46 and gp47, and DNA ligase (gp30). Such a combination of proteins suggests a minimal system for recombination in which nucleases 46/47 might generate ssDNA substrates suitable for homologous recombination by UvsX/gp41. The nicked DNA would then be sealed by DNA ligase. However, whether such a system is used is not known.

Unexpectedly, RNA-seq indicated that the expression of two late genes, *t* (holin, map units 160,221 to 160,877) and *38* (catalyzes assembly of distal tail fibers, map units 159,649 to 160,200), increased in the T4*motA^am^* infection (Figure 3). We confirmed increased levels for both by RT-qPCR (Figure S5a). Given the significantly reduced levels of expression of T4 genes *45* and *33*, whose products are required for late transcription, this result suggests an unusual expression mechanism for these late genes.

### 3.5. Reduction of Early Promoter Activity during T4 wt Infection in the Presence of DksA

Having established that the RNA analyses of T4 wt vs. T4*motA^am^* infections yielded expected results, we investigated gene expression in T4 infections of B606 wt or B606 ∆*dksA* to ask whether DksA reduction of T4 progeny is related to T4 gene expression.

In a T4 wt infection at 5 min, the absence of DksA resulted in a 2-fold or greater increase in expression of genes downstream of several Pe’s: Pe11.5, Pe12.8, Pe15.0, Pe40.4, Pe57954, Pe57.9, Pe69.9, and Pe69.4 (Figure 3). Only a small subset of the genes in these regions have been functionally characterized*.* The genes that have been characterized to date have been implicated in host takeover through modification of transcriptional machinery (*modA*, *modB*, *srh*, *mrh*) or degradation of host nucleic acid (*mobD*, *nudE*) [53]. RNA-seq was performed for the 5 min samples and RT-qPCR was used to independently assess the differential expression of 49 selected genes. The fold changes determined by RT-qPCR demonstrated a strong correlation with those determined by RNA-seq, thus confirming results of the RNA-seq analysis (Figure S5b). In addition, primer extension analyses were performed for RNA isolated at 1, 5, and 12 min post-infection. Primer extension analyses also revealed a significant increase in transcription from Pe35.3, Pe40.4, and Pe41 (Figure S6b). In contrast, transcription from Pm’s was unaffected or only very modestly affected (Pm*45*, Pm*45.2*) by the absence of DksA (Figure S6a). The increase in Pe transcription was not general. For example, no significant increase was observed for Pe148.6 (Figure S6b) and Pe144.6 (Figure S7), two Pe’s whose downstream gene expression levels also did not increase in the RNA-seq analysis (Figure 3).

Somewhat surprisingly, the expression level of several late genes (Figure 3) and the level of transcription from the corresponding Pl’s (Figure S7a) was significantly less at 5 min post-infection in the absence of DksA. Since this result is not compatible with an increase in burst size, we asked whether this decrease in late transcription was reversed at 12 min post-infection, when the level of late transcription in a wt infection is high. Primer extension analyses revealed that the level of RNA from these and other selected late promoters recovered well by this time point (Figure S7b).

Taken together, our results are consistent with a role of DksA in lowering the burst of T4 wt phage in a wt infection by decreasing the level of transcription from many Pe’s. As mentioned above, the function of many early genes remains unknown. However, these early genes are predicted to be involved in host takeover and transition to middle gene expression [53]. It is plausible that a fitness gain early in infection will cascade to increased progeny production as the infection progresses.

### 3.6. Middle Gene Expression is Partially Restored in a T4motA^am^ Infection of B606 ∆dksA

The absence of DksA alleviated the poor growth of T4*motA^am^* by significantly decreasing the latent period (Figure 2). To determine if DksA affects gene expression in the T4*motA^am^* infection, RNA-seq, RT-qPCR, and primer extension analyses were performed after isolating RNA from T4*motA^am^* infections in the presence (B606 wt) or absence (B606 ∆*dksA*) of DksA.

The overall result of these analyses is that the DksA deletion partially ameliorates the poor expression of many T4 middle genes through the increase in transcription from certain Pe’s. This result is most easily seen in Figure 3, in which expression levels of T4 genes are color coded to designate the extent of increase (shades of red) or decrease (shades of green). For example, in regions whose pre-replicative RNA levels were severely depressed in the T4*motA^am^*/wt infection (the region containing genes *43*, *44*, *62*, and *45* (map units 32,662 to 27,197) and the region containing genes *33*, *rnh*, *59*, and *32* (map units 150,727 to 146,948)), the level of RNA from the T4*motA^am^* infection in the absence of DksA increases from 1.5 to 7-fold over that seen in the T4*motA^am^* infection in the presence of DksA. These are the middle genes that are most severely affected by the *motA* knock-down.

Because the absence of DksA also increases the level of *motA* transcription in the *motA^am^* background by ~2-fold (Figure 3) and the *motA^am^* mutation is leaky, some of this increase could arise from an increase in MotA protein, which could then lead to more Pm activation. However, it might also arise from the increase in transcription from Pe’s, which then extends into downstream middle genes. To observe early and middle transcription separately, we used primer extension analyses.

We found that for the region containing map units 35,014 to 30,342 increased transcription from Pe35.3 in the absence of DksA was particularly helpful in counter-acting the effect of the *motA* knock-down since we observed no significant increase in middle transcription from Pm*46* or Pm*45* (Figures S6 and S8). Likewise, in the *rnh*-*32* region (map units 150,727 to 146,948), we observed that the overall increase in transcription resulted from a significant increase in transcription from Pe148.6 rather than an increase in Pm*dsbA* (Figure 4 and Figure S6b). In this case, a low level of Pe148.6 RNA was observed in the T4*motA^am^* infection of B606 wt at 5 min (Figure 4). However, the level of Pe148.6 RNA returned to that seen in the T4 wt/B606 wt infection when the *motA* knockdown was combined with the absence of DksA (Figure 4 and Figure S6b).

Unlike the middle genes detailed above, the transcript level of gene *55* (late sigma factor; map units 40,157 to 39,600) was not significantly reduced by the *motA* knockdown, yet gene *55* expression increased even more in a T4*motA^am^* infection in the absence of DksA (Figure 3). Primer extensions indicate that increased transcription of *55* is coming from Pe40.4 rather than from Pm*55* (Figures S6, S8c and S8d). In addition, primer extensions revealed that Pe40.4 RNA is processed at map unit 40,902. This position contains the consensus sequence recognized by RegB, a T4-encoded sequence-specific endonuclease that inactivates T4 early transcripts shortly after infection [59,60]. The transcript levels near (Figure S6b) and downstream (Figure S8d) from Pe40.4 were similar, indicative of regulation at or near the promoter either directly or indirectly by DksA.

No novel primer extension products were observed in any of the tested regions in the absence of DksA (Figure 4 and Figure S8), indicating that the deletion of DksA does not generate new transcription starts. As genes in regions transcribed by Pe35.3 (map units 35,662 to 27,197), Pe40.4 (map units 41,225 to 39,600), and Pe148.6 (map units 150,727 to 146,948) are required for replication and late transcription, it is not surprising that an increase in transcription from these promoters in the absence of DksA would improve the growth of the T4*motA^am^* phage in B606 ∆*dksA* relative to B606 wt.

### 3.7. DksA Does Not Inhibit T4 Pe Transcription In Vitro

**Figure 5:**
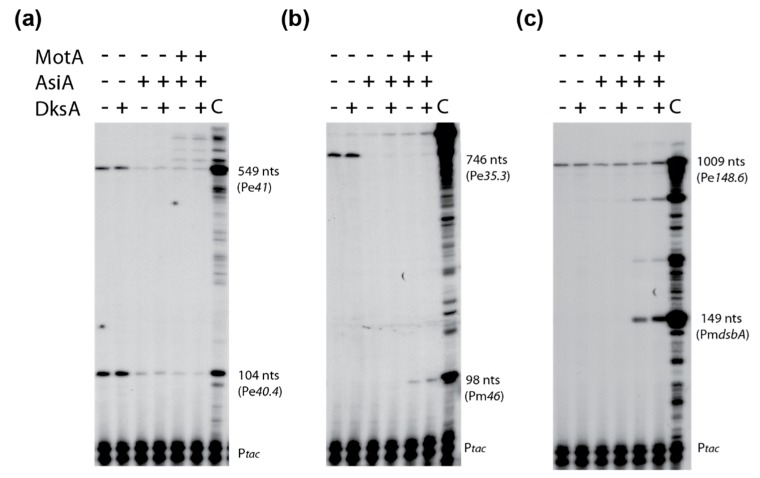
Presence of DksA has no effect on transcription from various T4 Pe’s in vitro. Representative gels show primer extension products for Pe40.4 (**a**), Pm*46* (**b**), and Pm*dsbA* (**c**) arising from in vitro transcription of T4 wt DNA in the presence of DksA, MotA, and/or AsiA, as indicated. Each primer extension reaction also contained P*tac* RNA and its primer as an internal control. In vivo RNA isolated from a T4 wt infection of B606 wt at 5 min post-infection was used as a positive control for each primer (labeled as lane C). The lengths of primer extension products are indicated in nt.

Our results suggested that host protein DksA might directly down-regulate the activity of T4 Pe’s. Although DksA regulated promoters often have AT- or GC-rich sequences within the −4 to −6 region (called the discriminator) that are needed for DksA action [61,62], a comparison of the sequences of down-regulated and unchanged Pe’s revealed no obvious sequence motif that typically correlates with DksA modulation of transcription (Patterson-West and Hinton. NIDDK, Bethesda, MD. Comparison of promoter sequences, 2017.). To ask whether DksA inhibited Pe transcription directly, we first purified DksA and tested its previously characterized activity to inhibit the ribosomal promoter P*rrnB1*. As seen in Figure S9, addition of DksA specifically inhibited P*rrnB1* but did not affect transcription from a control σ^70^-dependent promoter (P*S17*). As expected, the presence of ppGpp improved this inhibition. Next, we performed in vitro transcription reactions using T4 DNA under the same buffer conditions. We assayed for transcription from specific Pe’s using primer extension (Figure 5). DksA did not inhibit transcription from any of the tested Pe’s that we previously showed were affected by the presence of DksA in vivo: Pe40.4 or Pe41 (Figure 5a), Pe35.3 (Figure 5b), or Pe148.6 (Figure 5c). The presence of DksA also did not affect transcription from the middle promoters Pm*dsbA* (Figure 5c) or Pm*46* (Figure 5b). It is important to note that the overall level of MotA/AsiA-activated transcription in this in vitro system was low because of the use of buffer conditions suited for DksA. We speculate that DksA reduction of specific Pe activity is either indirect or requires additional factors/conditions that were not present in our in vitro transcription system.

### 3.8. Deletion of ppGpp Has Only Modest Effects on T4 Transcription

Although the absence of either DksA or ppGpp results in larger T4 plaques (Figure 1a and Figure S2b), the lack of DksA increases burst size by 2-fold (Figure 2b) while the absence of ppGpp has no significant effect on burst size or latent period (Figure S4). Our RNA-seq analyses of a T4 wt infection of a B606 ppGpp^0^ strain revealed only eight T4 genes whose expression changed significantly, while in the T4/∆*dksA* infection, the expression of >50 genes were altered (Tables S1 and S2).

## 4. Discussion

DksA, a transcriptional regulator that modulates gene expression in *E. coli* at the levels of transcription initiation [63] and elongation [15,64], belongs to a family of proteins that can insert themselves into the 2° channel of RNAP, the channel from which rNTPs enter the active site. The best characterized system of DksA regulation is stress response: the repression of ribosomal promoters and the activation of promoters for amino acid biosynthesis genes during amino acid starvation [65]. Both inhibition and activation typically involve the small molecule ppGpp [21], whose level rises dramatically during stress response, even as the level of DksA remains relatively constant [13,14]. For instance, the presence of ppGpp significantly improves the ability of DksA to inhibit initiation from ribosomal promoters [13].

DksA is a key player in bacterial survival under various environmental changes. For instance, ∆*dksA Salmonella enterica* is hyper-susceptible to the bacteriostatic effects of nitric oxide free radicals and is attenuated in macrophage and murine models of infection [66,67]; ∆*dksA Shigella flexneri* has decreased Hfq transcription, causing the loss of virulence [68]; and in *E. coli*, DksA has been shown to be important for survival of dehydration [69]. More recently, various studies have shown that DksA provokes global changes in transcriptional expression in host cells under various stresses, such as nitrosative, oxidative and nutrient stresses [70,71].

To our knowledge, no study has reported whether DksA contributes defense systems by modulating gene expression of phages during infection. Bacteriophage T4 is a good model system for investigating this since it primarily regulates gene expression at the level of transcription. Temporal gene expression by T4 is controlled by the recognition of three distinct promoter architectures (early, middle, and late). Pe’s contain very strong σ^70^ consensus sequences (−10 and −35 elements) that are immediately recognized by the host transcriptional machinery [23,72]. Pm’s and Pl’s require viral-encoded factors to modify the sequence specificity of host RNAP. Middle genes are expressed approximately 1 to 2 min post-infection at 37 °C by the activation of Pm’s and/or extension of transcripts from Pe’s (reviewed in [23]). Activation of Pm’s requires two T4 early proteins, MotA and AsiA, which together modify the specificity of host RNAP/σ^70^ holoenzyme, allowing it to recognize a specific −30 sequence (the MotA box) rather than the host −35 element. Late transcription is initiated approximately 5–7 min after infection by the activation of Pl’s. These promoters do not contain either the bacterial −35 element or the MotA box sequence, but instead contain a novel −10 sequence, known as the late TATA box, that requires a new sigma factor encoded by T4 gene *55*, as well as the activator encoded by gene *33* [24]. In addition, late transcription requires active T4 DNA replication through the activity of the T4 protein gp45, which is the replication sliding clamp that also serves as an enhancer of late transcription [24].

The major finding of this study is that host protein DksA down-regulates the activity of Pe’s, thereby limiting the number of progeny produced during a single infection. Consequently, DksA serves to suppress T4 infection. Increased transcription from specific Pe’s in the ∆*dksA* strain also explains the increased growth of T4*motA^am^* (Figure 1b). It has been well established that T4*motA^am^* severely impairs phage infection by depressing middle transcription. We observe that the improved fitness of T4*motA^am^* in the ∆*dksA* strain correlates with a greater abundance of middle RNA. However, this increase in middle transcripts does not arise from Pm’s, but rather from increased transcription from Pe’s upstream of middle genes required for replication, recombination, and late transcription (Figure 3). Thus, the increase in Pe transcription compensates for the lack of Pm activation.

Presently, it is unclear how DksA exerts an effect on specific Pe’s. The results of our in vitro transcription analyses argue that this effect is probably indirect. A comparison of the discriminator region [61,62] of Pe’s whose transcripts were down-regulated vs. those whose transcripts were unchanged does not reveal an obvious sequence motif that typically correlates with DksA reduction of promoter activity. We speculate global host changes that arise from deletion of DksA are likely to be responsible for the enhancement of T4 Pe transcription. In fact, global changes in gene expression during exponential growth have been previously reported for ∆*dksA* [21]. It is important to note, however, that we do not observe a major growth defect in B606 ∆*dksA* during early-exponential phase at the time of T4 infection. Instead the growth defect is seen during stationary phase (Figure S3). This is likely due to decreased *rpoS* expression in the ∆*dksA* mutant [73], which regulates expression of genes important for the transition into stationary phase.

Although the absence of either DksA or ppGpp results in larger T4 plaques (Figure 1a and Figure S2b), the lack of DksA increases burst size by 2-fold (Figure 2b) and has dramatic effects on T4 gene expression, while ppGpp^0^ has no significant effect on burst size or latent period (Figure S4) and has only modest effects on gene expression (Table S2). In the host, ppGpp functions predominately at the level of transcription to adjust gene expression as necessary to overcome stress and to minimize processes that have become superfluous by growth inhibition. Cells that are ppGpp deficient for lack of *spoT* and *relA* genes become vulnerable to a variety of physiological stress conditions. As a result, ppGpp^0^ cells devote all resources to a full complement of biosynthetic capacity including ribosome content regardless of nutritional adequacy [74]. Since T4 phage infection almost instantly pirates cellular biosynthetic functions and components to devote them exclusively to phage production, perhaps it can be expected that phage development would improve in the absence of ppGpp. Deficiency of ppGpp could alter plaque size without altering burst size, latent period, and/or transcription because plaque size is also governed by properties of inner membranes, peptidoglycan and outer membranes, and the activities of pinholins and spanin complexes (reviewed by [75]). In fact, there is evidence of involvement of ppGpp in altered membranes and phospholipid synthesis that can result in membrane fragility [76,77].

The enigmatic absence of regulatory effects associated with ppGpp might indicate the need for different conditions to detect effects of ppGpp on T4 burst size and latent period. For example, slow growth in minimal medium with a poor carbon source, which elevates the level of ppGpp, might give different results than a rich nutrient broth where the cellular ribosomal content is high and the ppGpp concentration is very low at the onset of infection. It also might be revealing to measure ppGpp levels during phage T4 lytic development to evaluate if ppGpp levels change. Alternatively, ppGpp levels could be artificially elevated to high levels at the onset of T4 infection to ask if development is altered.

Host factors that regulate viral transcription during phage infections have been reported for multiple systems. In several cases, phage-encoded proteins that modulate the functions of these bacterial regulators have also been observed. These include the *E. coli* transcription terminator Rho and lambdoid phage antiterminators, N and Q (reviewed in [78]). In addition, bacterial histone-like proteins, such as H-NS in *E. coli*, can repress viral transcription (reviewed in [79]). Interestingly, the T7 protein gp5.5 [80], the T4 protein Arn [81], and the phage LUZ24 protein MvaT [82] have each been shown to inhibit H-NS repression through distinct mechanisms. Future work investigating the effects of bacterial transcriptional regulators will likely reveal additional host proteins that regulate viral gene expression. Understanding the role of ppGpp, DksA, and other *E. coli* factors that regulate transcription and transcription/translation coupling, such as GreA and NusG, may reveal new mechanisms required for host takeover and identify new functions of additional viral proteins.

## Supplementary Materials

The following are available online at http://www.mdpi.com/1999-4915/10/6/308/s1, Figure S1: DksA-His_6_ is highly purified, Figure S2: T4 infections of B606 ppGpp^0^ produce larger plaques, Figure S3: Minor growth differences between wt and mutant strains during early exponential phase, Figure S4: Absence of ppGpp does not affect burst size or latent period of a T4 wt infection, Figure S5: There is a strong correlation between the relative level of RNA determined by RNA-seq and RT-qPCR, Figure S6: The absence of DksA increases the level of transcription from T4 Pe’s in a T4 wt and a T4*motA^am^* infection at 5 min post-infection, Figure S7: Transcription from late promoters is lower in the absence of DksA (∆*dksA*) compared to B606 wt at 5 min post-infection but returns to values near B606 wt levels by 12 min post-infection, Figure S8: Increased levels of middle genes *46* and *55* in T4*motA^am^* infections of cells lacking DksA (∆*dksA*) are due to increased levels of transcripts from an upstream early promoter at 5 min post-infection, Figure S9: Purified DksA inhibits P*rrnB1* transcription in vitro, Table S1: Summary of all RNA-seq and RT-qPCR data, Table S2: Summary of genes that are differentially expressed in T4 wt infections of ∆*dksA* and ppGpp^0^ compared to wt *E. coli.*
