# Supplementary material for: The E. coli Global Regulator DksA Reduces Transcription during T4 Infection
Source: Viruses. 2018 Jun 6;10(6):308. doi: 10.3390/v10060308 (PMC6024815; doi:10.3390/v10060308)
Supplement: Supplementary file 1 [file viruses-10-00308-s001.zip › viruses-292162-supplementary/viruses-292162-supplementary_Final.docx]

**Supplemental Material**


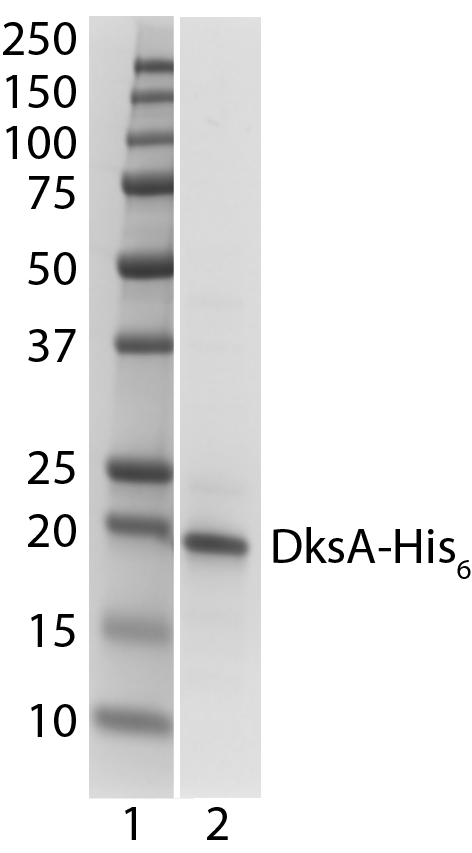


**Figure S1. DksA-His_6_ is highly purified.** An aliquot of DksA-His_6_ (250 mM eluent, lane 2) after Ni^++^ affinity chromatography was electrophoresed on a SDS-PAGE gel and stained with Coomassie. Lane 1 contains Precision Plus Protein standard (Bio-Rad); the sizes of the standards are indicated.


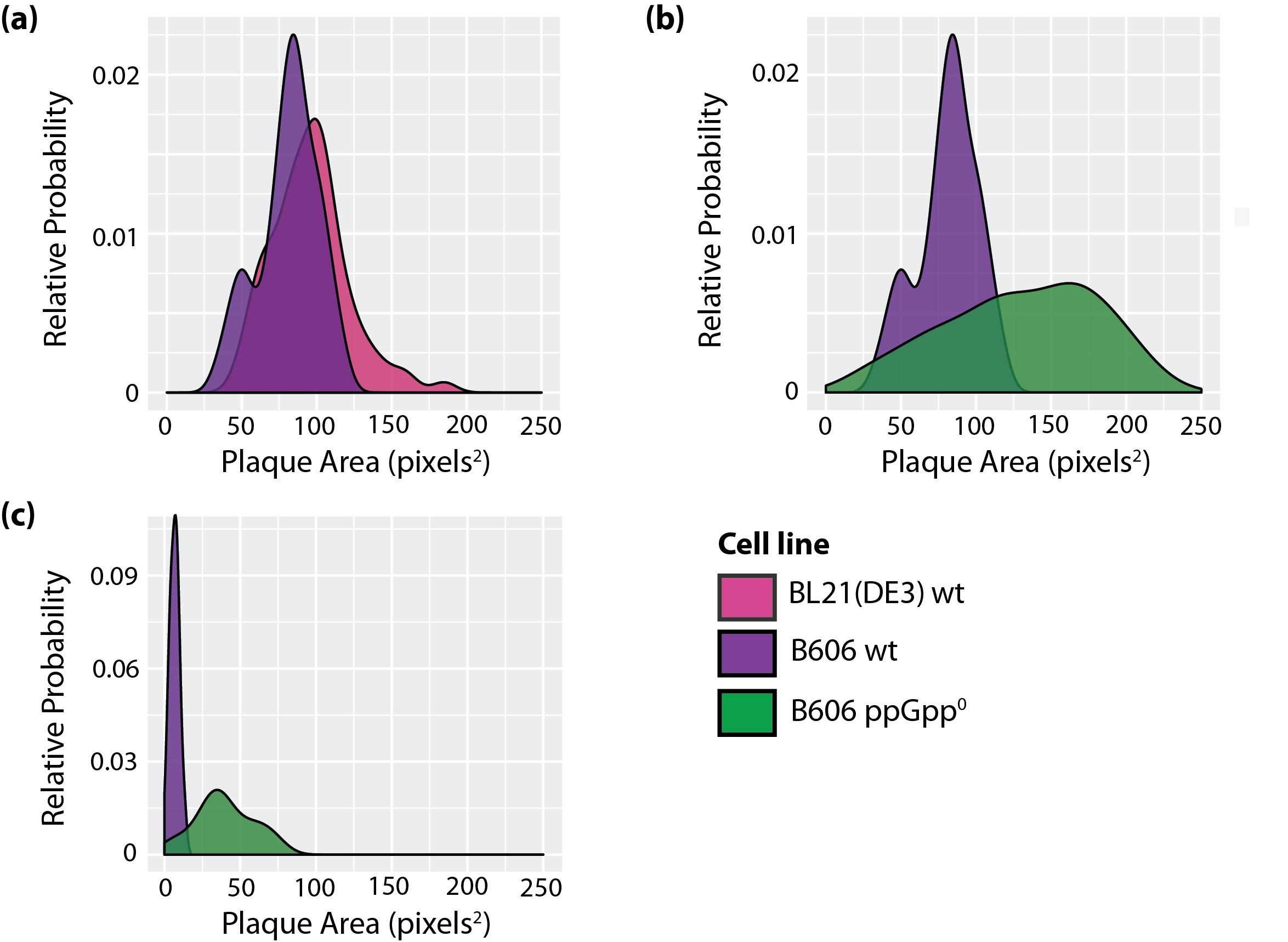


**Figure S2. T4 infections of B606 ppGpp^0^ produce larger plaques.** Representative density plot of plaque size: (**a**) T4 wt infection of BL21(DE3) wt (pink) and B606 wt (purple); (**b**) T4 wt infection of B606 wt (purple) and B606 ppGpp^0^ (green); and (**c**) T4*motA^am^* infection of B606 wt (purple) and B606 ppGpp^0^ (green). Plots shown are from one of three biological replicates.


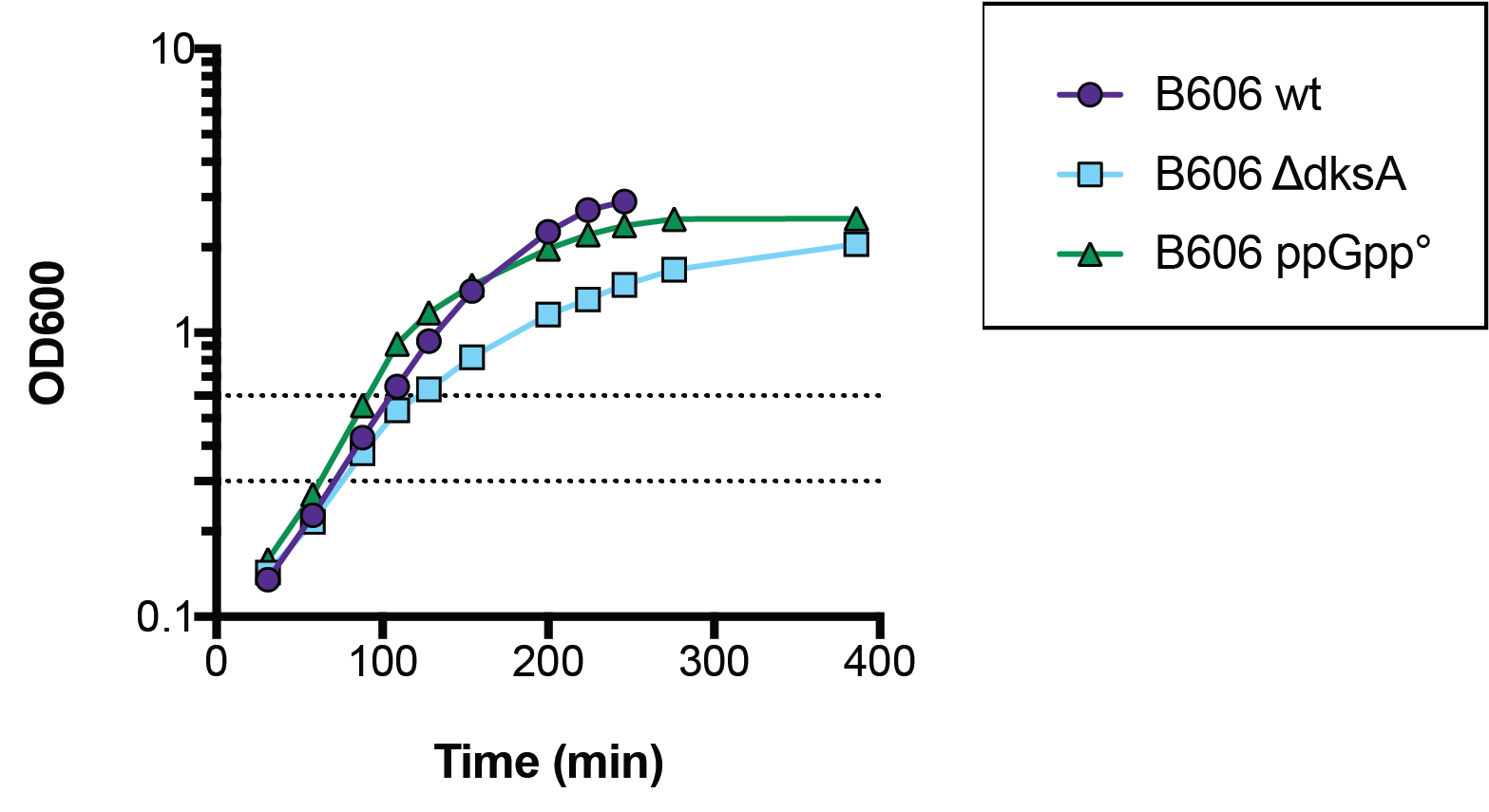


**Figure S3. Minor growth differences between wt and mutant strains during early exponential phase.** Representative growth curves for B606 wt (purple), B606 Δ*dksA* (Carolina blue), and B606 ppGpp^0^ (green). The growth curve during early exponential phase is highlighted by dotted lines at OD_600_ 0.3 and 0.6 to illustrate that minor differences are observed during this growth phase, when T4 infections are performed.

**
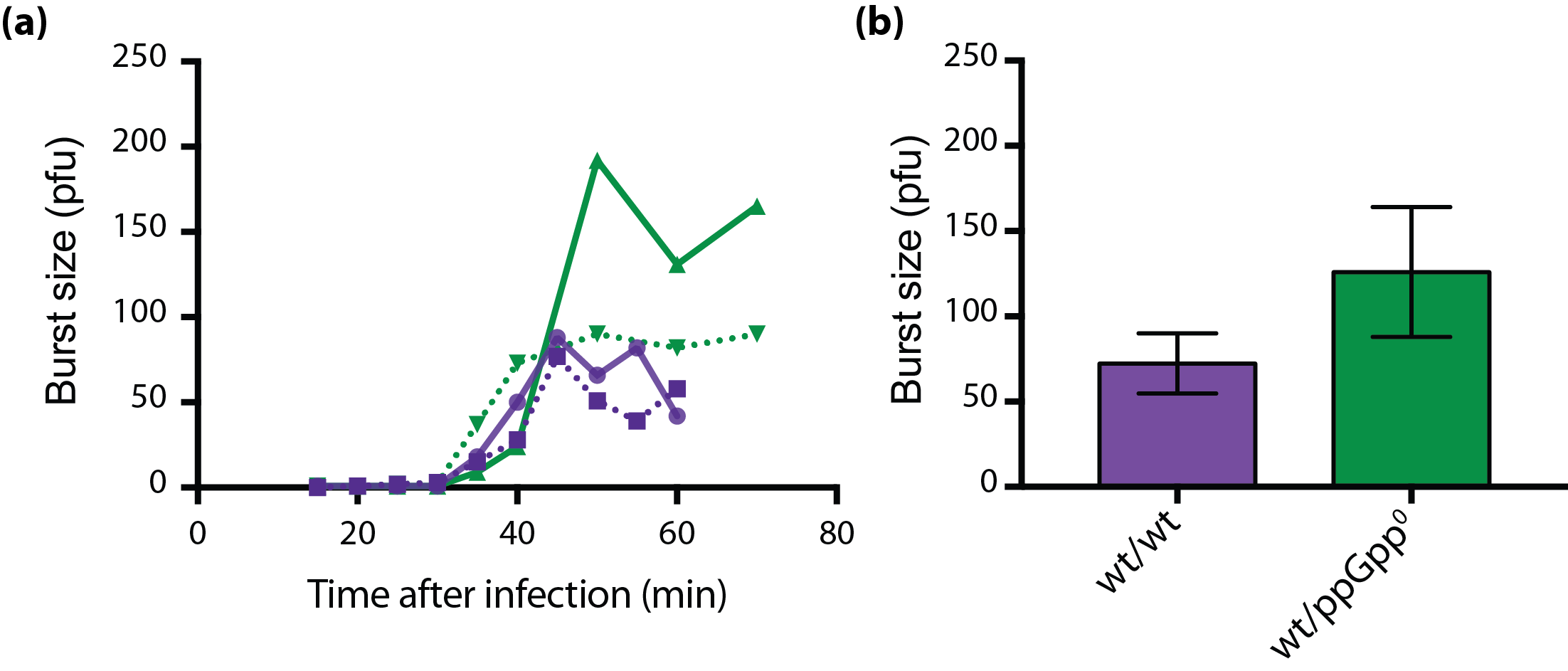
**

**Figure S4. Absence of ppGpp does not affect burst size or latent period of a T4 wt infection.**  Representative graph of the burst size vs. time after infection (**a**) and burst size (**b**) for infections of T4 wt/B606 wt (purple) and T4 wt/B606 ppGpp^0^ (green). The solid and dashed lines show data from two independent replicates to illustrate the variability in the burst size for T4 wt infections of ppGpp^0^. Averages with indicated standard deviations were determined as described in Materials and Methods from three independent replicates. There is no significant difference in the burst size between wt/wt and wt/ppGpp^0^ based on overlapping standard deviations in (b).

**
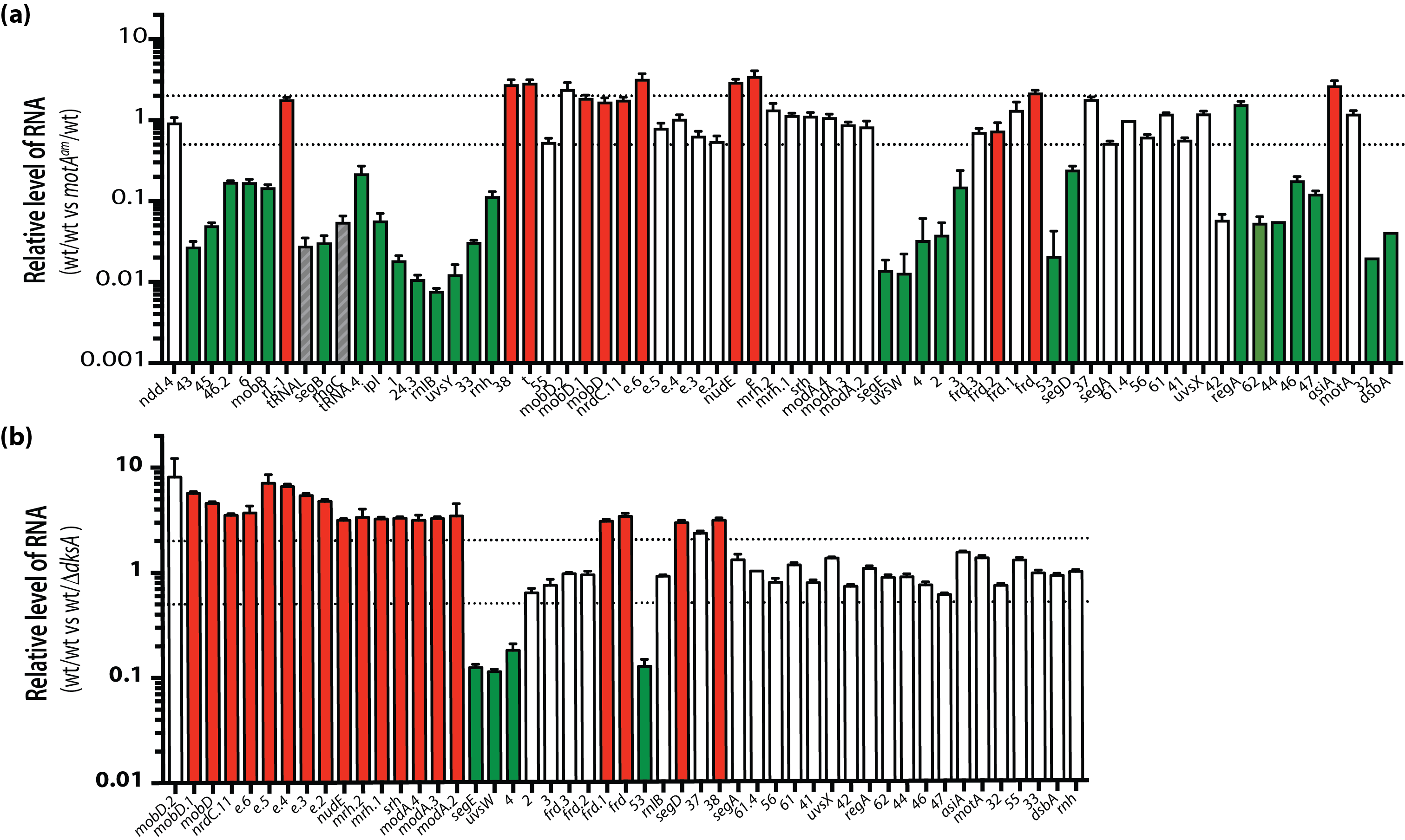
**

**Figure S5. There is a strong correlation between the relative level of RNA determined by RNA-seq and RT-qPCR.** Bar plots show the level of RNA of various T4 genes determined by RT-qPCR for a T4 wt/B606 wt (wt/wt) vs. a T4*motA^am^/*B606 wt (*motA^am^/*wt) infection (**a**) and for a T4 wt/B606 wt (wt/wt) vs. a T4 wt/B606 Δ*dksA* (wt/Δ*dksA*) infection (**b**). Values for RT-qPCR are based on the average of three biological replicates and two technical replicates of each. Bars are colored according to the relative level of RNA determined by RNA-seq: red, increasing expression ≥ 2.0; white, no significant change; green, decreasing expression ≤ 0.5; and grey, less than 50 mapped reads in all the RNA-seq data samples. Dotted lines indicate the threshold for a 2-fold change in the level of RNA.


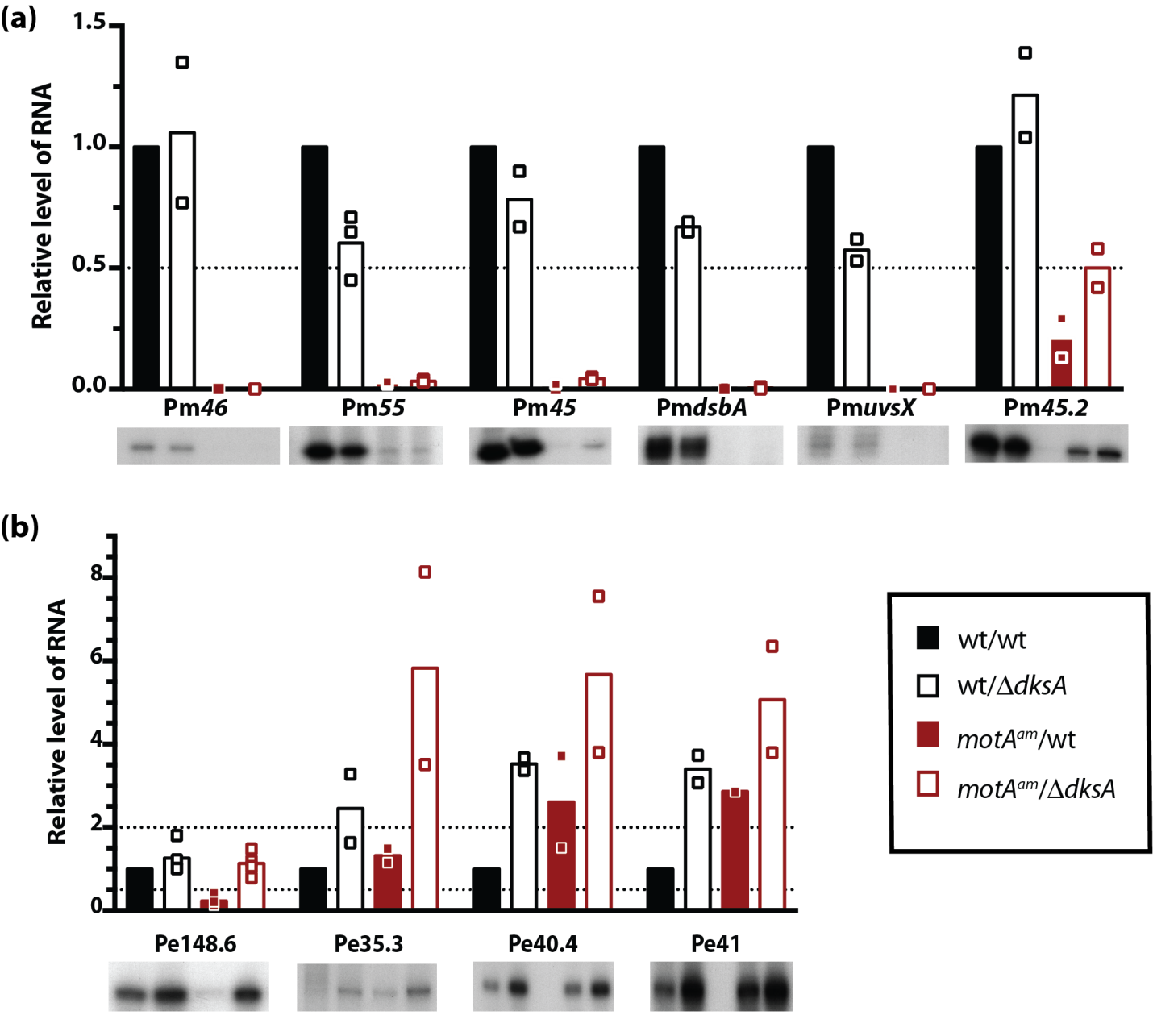


**Figure S6. The absence of DksA increases the level of transcription from T4 Pe’s in a T4 wt and a T4*motA^am^* infection at 5 min post-infection.** Histograms show the determined levels of RNA from indicated middle (**a**) or early (**b**) promoters. Primer extension products corresponding to each indicated promoter are shown below from a representative gel. Primer extensions were repeated using at least two biological replicates. The relative level of RNA for each replicate is shown as a black open square (wt/Δ*dksA*), a red solid square with white outline (*motA^am^/*wt), or a red open square (*motA^am^/*Δ*dksA*). The dotted line indicates the threshold for a 2-fold change in the level of RNA. No product was detected for Pm*46* or Pm*uvsX* in either T4*motA^am^*/B606 wt or *motA^am^*/B606 Δ*dksA* infections at 5 min post-infection.

**
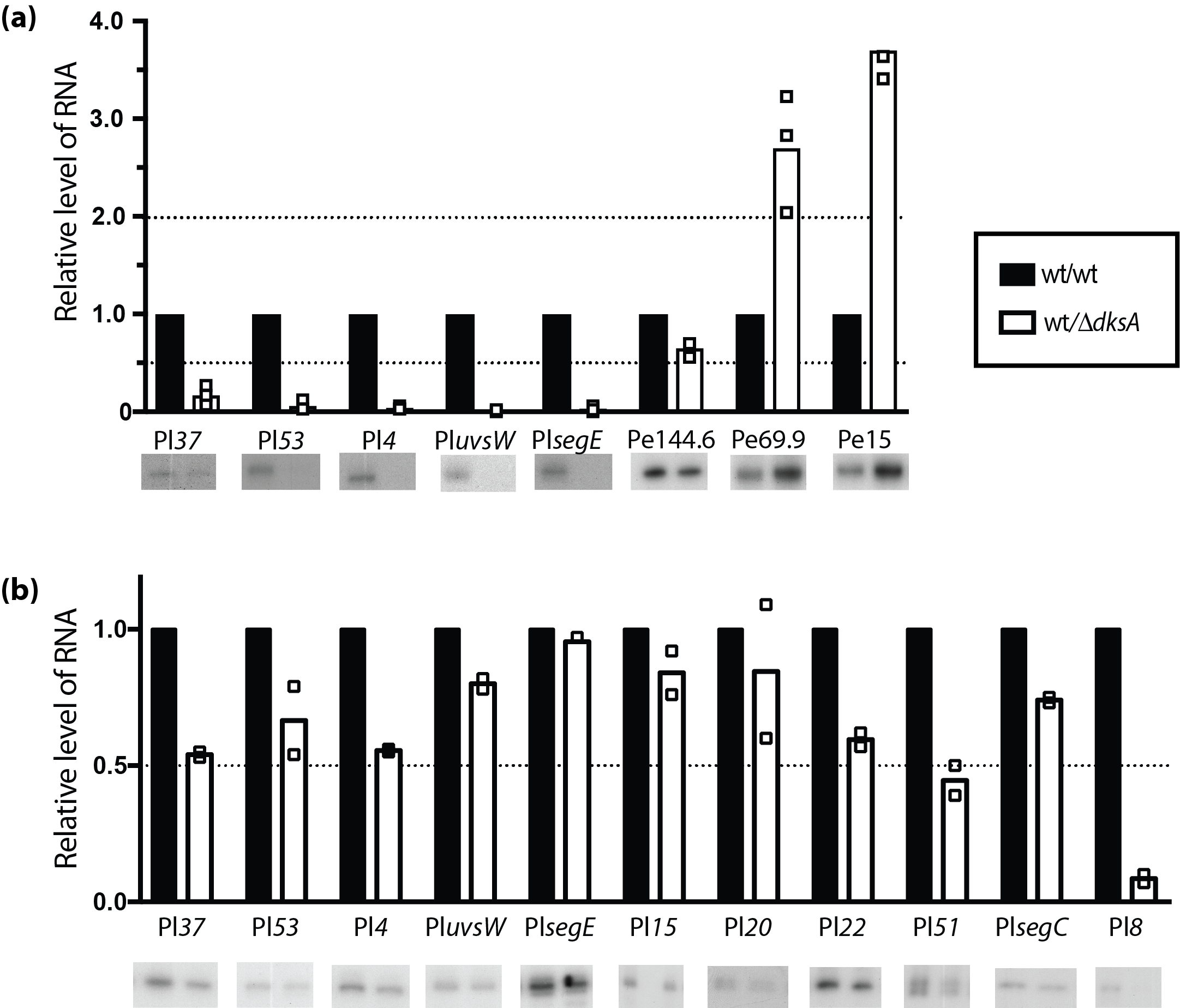
**

**Figure S7. Transcription from late promoters is lower in the absence of DksA (Δ*dksA*) compared to B606 wt at 5 min post-infection but returns to values near B606 wt levels by 12 min post-infection.** Histograms show the relative level of RNA from various late promoters in the indicated infections: solid black, T4 wt infection of wt B606 (wt/wt) and open black, T4 wt infection of B606 Δ*dksA* (wt/Δ*dksA*) at 5 min (**a**) or 12 min (**b**) post-infection. The bands corresponding to each promoter are shown below from a representative gel. All primer extensions were repeated using at least 2 biological replicates. The relative level of RNA for each replicate is shown as an open black square. Note that the two replicates for Pl*4* shown in (**b**) overlap to an extent that they appear as a solid square. The dotted lines indicate the threshold for a 2-fold change in the level of RNA. We also show a set of primer extensions for early promoters at 5 min (a) post-infection to illustrate that transcript levels from these promoters are either unaffected or upregulated.

**
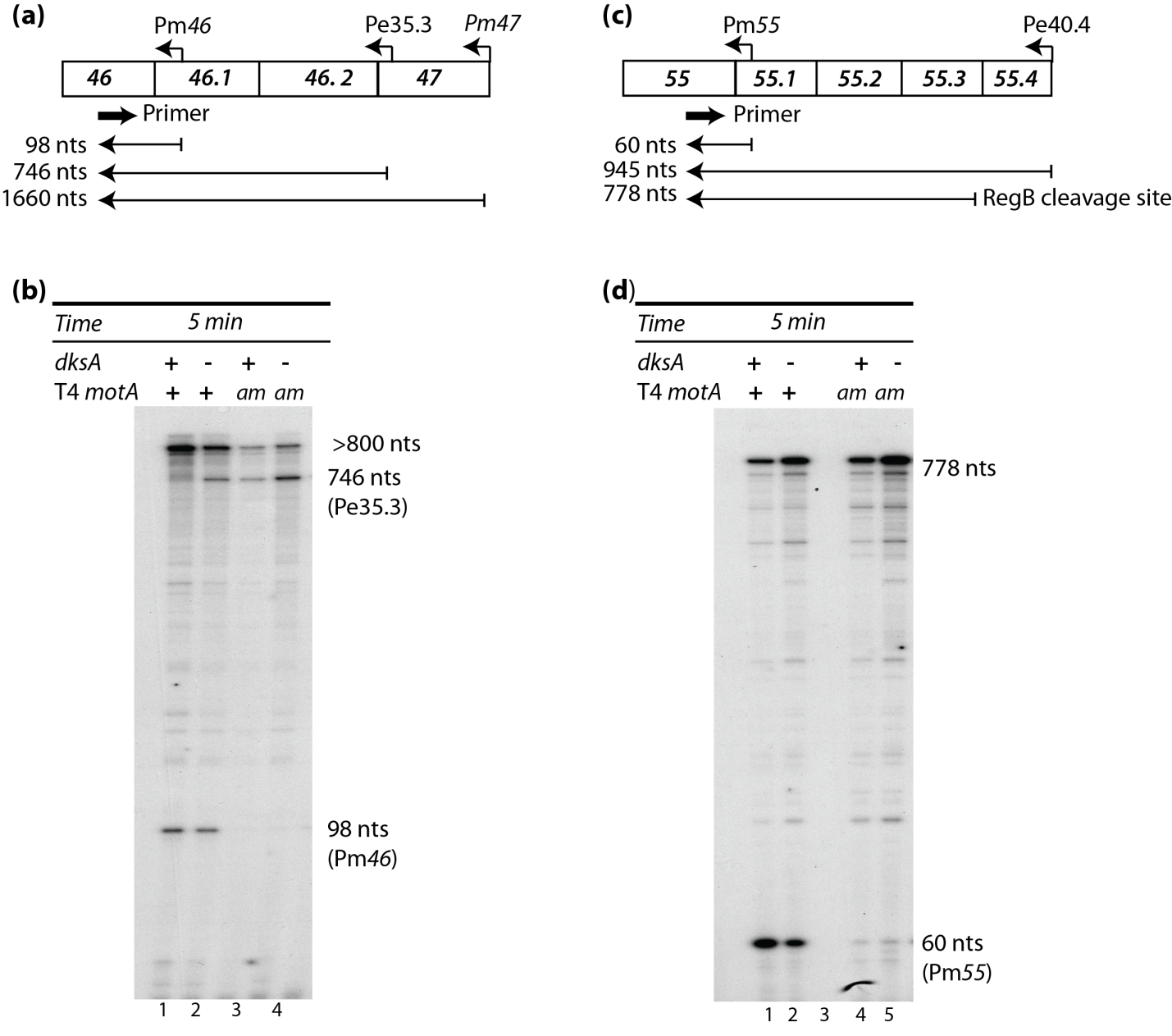
**

**Figure S8. Increased levels of middle genes *46* and *55* in T4*motA^am^* infections of cells lacking DksA (Δ*dksA*) are due to increased levels of transcripts from an upstream early promoter at 5 min post-infection.** Schematics of the T4 genomic map from 33,302-36,576 (**a**) and 39,600-41,225 (**c**) showing previously identified promoters and relevant transcripts with lengths specified [53]. Representative gels show primer extension products from primers in genes *46* (**b**) and 55 (**d**). RNA was isolated from T4 wt (+) or T4*motA^am^* (*am*) infections of the indicated *E. coli* strain B606 wt (+) or B606 Δ*dksA* (-) at 5 min post-infection.


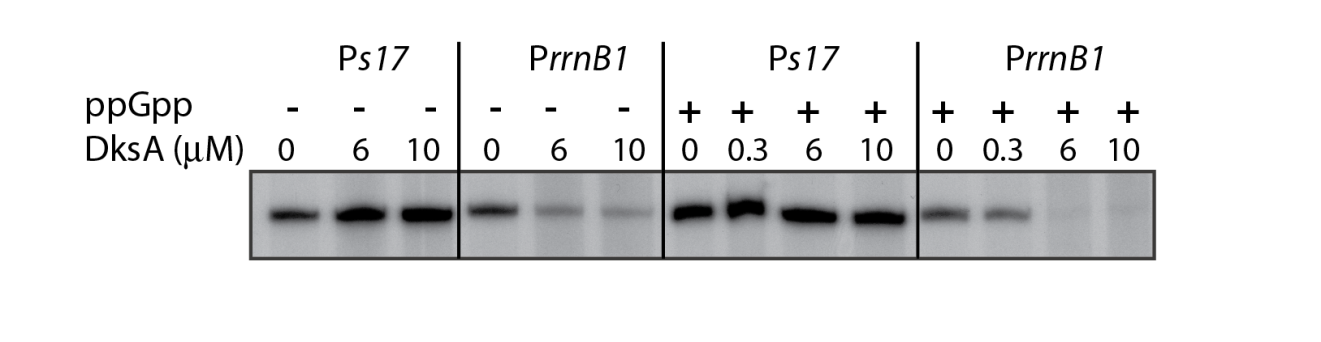


**Figure S9. Purified DksA inhibits P*rrnB1* transcription *in vitro*.** Representative gel slice showing radiolabeled RNA from ribosomal P*rrnB1* promoter, which is inhibited by DksA (positive control) or a σ70-dependent promoter P*S17*, which is not inhibited by DksA (negative control). Transcriptions were performed in the presence (+) or absence (-) of 250 μM ppGpp with the indicated amount of DksA.

**Table S1.** **Summary of all RNA-seq and RT-qPCR data.** RNA-seq count table for T4 wt and T4*motA^am^* infections of B606 wt, Δ*dksA*, and ppGpp^0^ are listed. Gene expression changes are color coded as: increasing expression ≥ 2.0 with increasing red intensity; no significant change (white); decreasing expression ≤ 0.5 with increasing green intensity. Values of genes whose changes in gene expression were verified by RT-qPCR are given. Fold changes that were not consistent between RNA-seq and RT-qPCR are given in red. (Excel file)

**Table S2. Summary of genes that are differentially expressed in T4 wt infections of Δ*dksA* and ppGpp^0^ compared to wt *E. coli*.** The mean count from RNA-seq for T4 wt infections of B606 wt, Δ*dksA*, and ppGpp^0^ are listed. Fold changes for differentially expressed genes are color coded as: increasing expression ≥ 2.0 with increasing red intensity or decreasing expression ≤ 0.5 with increasing green intensity. The Benjamini-Hochberg adjusted *p* value is listed for each gene.

| **Gene** | **Mean Counts** | | | **wt/wt vs. Δ*dksA*/wt** | | **wt/wt vs. ppGpp^0^/wt** | |
| --- | --- | --- | --- | --- | --- | --- | --- |
|  | **wt** | **Δ*dksA*** | **ppGpp^0^** | **Fold**  **change** | ***p* value** | **Fold**  **change** | ***p* value** |
| *4* | 527 | 36 | 272 | 0.11 | 1.40E-13 | 0.39 | 3.64E-03 |
| *uvsW* | 2297 | 297 | 1391 | 0.2 | 6.99E-15 | 0.45 | 2.88E-04 |
| *mobD.1* | 1293 | 2278 | 3228 | 2.87 | 8.70E-12 | 2.07 | 9.00E-06 |
| *nrdC.11* | 8568 | 16584 | 23715 | 2.92 | 4.30E-91 | 2.19 | 9.80E-49 |
| *srh* | 538 | 1098 | 1405 | 3.03 | 1.40E-23 | 2.05 | 4.50E-10 |
| *mobD* | 3765 | 7554 | 10452 | 3.14 | 4.80E-24 | 2.25 | 7.80E-12 |
| *e.2* | 802 | 2005 | 2261 | 3.51 | 1.50E-20 | 2.09 | 2.00E-07 |
| *e.4* | 522 | 1649 | 1434 | 4.57 | 1.20E-16 | 2.13 | 1.30E-04 |
